# Differential effects of simulated restorative environments on subjective and objective attentional outcomes

**DOI:** 10.3389/fpsyg.2026.1791175

**Published:** 2026-07-10

**Authors:** Elisabetta Ricciardi, Luigi Tinella, Rosa Napoletano, Sergio Traficante, Antonella Lopez, Alessandro Oronzo Caffò, Stefano Triberti, Andrea Bosco, Giuseppina Spano

**Affiliations:** 1 Department of Precision and Regenerative Medicine and Ionian Area, University of Bari, Bari, Italy; 2 Department of Psychology and Health Science, Pegaso University, Naples, Italy; 3 Department of Educational Sciences, Psychology, Communication, University of Bari, Bari, Italy; 4 Department of Human, Social and Educational Sciences, University of Molise, Campobasso, Italy; 5 LAHTI Laboratory for Advanced Human-Technology Interaction, Pegaso University, Milan, Italy

**Keywords:** artistic stimuli, attention, Bayesian analysis, objective cognition, simulated nature, subjective cognition

## Abstract

Virtual environments are increasingly used to promote cognitive and psychological well-being, yet the influence of environment type and user characteristics (e.g., age) remains unclear, particularly in low-immersion formats such as videos. Moreover, the relationship between subjective and objective measures of cognition warrants further investigation. This study examined the effects of brief exposure to simulated 2D natural, artistic, or control (office) environments on subjective Perceived Attentive Efficiency, objective Cognitive Performance, and Perceived Restorativeness in 88 participants (44 young, 44 older adults). Participants completed single-item ratings of perceived attentive efficiency and digitalized neuropsychological tasks pre- and post-exposure, while restorativeness was assessed only post-exposure. Bayesian repeated-measures ANOVAs showed that participants exposed to natural and artistic environments reported higher perceived attentive efficiency and restorativeness than those exposed to the control environment. However, no corresponding improvements were observed in the objective cognitive measures. These findings suggest that, under the present experimental conditions, brief exposure to simulated 2D environments was associated with changes in subjective but not objective attentional outcomes. The comparable pattern of results observed for natural and artistic environments further suggests that factors such as positive valence or aesthetic appeal may contribute to perceived benefits, although the specific mechanisms involved require further investigation. No age-related differences emerged, indicating broadly similar responses across younger and older adults. Overall, the findings highlight the importance of considering both subjective and objective indicators when evaluating restorative environmental interventions and provide preliminary support for the potential value of scalable, low-cost virtual experiences for promoting perceived cognitive and psychological well-being.

## 1 Introduction

Virtual Reality (VR) has emerged as a versatile tool in psychological research, offering unique opportunities to explore and manipulate various aspects of human behavior, emotion, and cognition (Frost et al., 2022; Li et al., 2021b; Spano et al., 2023). By immersing individuals in controlled virtual environments, researchers can systematically expose them to stimuli that trigger specific psychological responses, while offering a safe and controlled setting for therapeutic interventions and consistent exposure across participants in experimental cognition (White et al., 2018; Cipresso et al., 2018). Researchers are increasingly utilizing VR, both in 2D and immersive 3D formats, as a tool for promoting psychological well-being (Pasca et al., 2022). Evidence shows that the key factor contributing to these benefits is the immersive experience, often associated with its psychological counterpart or the “sense of presence,” i.e., the subjective experience of “being there” in the virtual environment, compared to traditional 2D media like photos and videos (Liszio et al., 2018; Li et al., 2021a; Yeo et al., 2020). On the other hand, recent research highlights that some determinant factors in the sense of presence are independent of mere immersion or technology sophistication (Triberti et al., 2025): for example, psychological aspects, such as consistency of the virtual experience with users’ intentionality or an engaging narrative context may contribute to promote sense of presence even within simulation with limited immersive properties. In any case, episodes of motion sickness in 3D environments, with symptoms such as nausea, dizziness, and disorientation, are a relatively common issue that affects susceptible individuals, including older adults (Howard and Van Zandt, 2021).

Age differences in responses to virtual environments are emerging as a critical area of investigation. Older adults often encounter barriers in accessing physical environments, such as nature or cultural spaces, due to physical or logistical limitations. Virtual environments present a promising solution for fostering cognitive and emotional engagement among older individuals. Despite potential challenges in navigating VR technologies, older participants may derive meaningful benefits in terms of improved mood and cognitive engagement from exposure to nature-based virtual environments (Kalantari et al., 2022).

Research suggests that older adults may be more susceptible to attentional fatigue due to age-related changes in attention and executive function, which could increase their need for environmental restoration. However, the specific mechanisms underlying age-related differences in restoration remain poorly understood, particularly in virtual environments, including both immersive 3D and simulated 2D formats, where the physical constraints that often limit older adults’ access to natural settings are removed.

### 1.1 Cognitive applications of ART theory and the problem of inconclusive evidence

Among the various settings that virtual reality can simulate, natural environments have received growing attention within the framework of Attention Restoration Theory (ART) (Kaplan, 1995). According to ART, natural environments possess four key restorative qualities: fascination (effortless attention), being away (mental distance from routine demands), extent (rich environments that engage the mind), and compatibility (environments that support one’s goals and inclinations). Most studies related to this research topic have examined the effects of virtual nature on perceived restorativeness. Studies highlight comparable effects between virtual and outdoor natural environments, especially regarding the fascination and the “being away” effects (Calogiuri et al., 2018; Reese et al., 2022). Furthermore, exposure to both virtual and outdoor natural environments predict greater restorativeness compared to indoor settings (Browning et al., 2020). Notably, urban environments, commonly used as a comparison environment, may be perceived as aversive, potentially biasing affective comparisons in favor of natural settings. Including other positively-valenced environments (e.g., cultural or artistic) could help clarify the specific restorative effects of nature.

Considerably less has been investigated regarding the effects of exposure to virtual nature on cognitive outcomes, and existing evidence depicts a complex picture. Overall, there do not appear to be significant effects of mere exposure on general cognitive functioning (Spano et al., 2023) nor on attention in both cases of videos featuring natural and urban environments (Yeo et al., 2020) and memory, comparing a virtual vs. outdoor nature walk (Léger and Mekari, 2022). However, opposing evidence is reported by a study (Mostajeran et al., 2023) indicating that exposure to virtual nature (i.e., a 3D forest environment) led to significantly higher cognitive performance, in terms of executive functions (using trail making tests A, B, and B-A) as well as memory (using digit span forward and digit span backward tests) compared to the control environment. Along the same lines, Berto (2005) supported the idea that viewing images of restorative environments helped previously stressed participants restore their performance on a sustained attention task. This inconsistency is also evident in studies examining the potential effect of in-vivo greenspace exposure on cognitive functioning (Ricciardi et al., 2022; Ricciardi et al., 2025), with findings ranging from mixed results to an absence of evidence across the literature to domain- and subgroup-specific effects, including positive associations for certain cognitive functions (e.g., language) and null associations for others (e.g., attention). These findings suggest that the relationship between environmental exposure and cognitive performance requires further investigation across different modalities of experience.

An important consideration that has received limited attention in virtual environment research, across immersive 3D and simulated 2D formats, is the potential discrepancy between subjective perceptions of cognitive improvement and objective performance measures. While participants may report feeling more alert, focused, or cognitively capable following exposure to restorative environments, these subjective improvements may not necessarily translate into measurable cognitive performance gains. This subjective–objective divide has significant implications for understanding the mechanisms underlying environmental restoration effects and their practical applications. The distinction between perceived and actual cognitive performance becomes particularly relevant when considering the self-regulatory aspects of attention and cognitive control. Individuals’ beliefs about their cognitive capabilities can influence their engagement with tasks and their overall well-being, independent of measurable performance improvements (Bandura, 1997). These beliefs represent a relatively stable, identity related evaluation of one’s abilities, which is distinct from the transient, context dependent experience of feeling restored by a given environment. Empirical evidence reinforces this subjective–objective dissociation: while exposure to urban green environments reliably enhances positive affect and perceived restorativeness (Barnes et al., 2019; Menardo et al., 2021; Olszewska-Guizzo et al., 2022; Yao et al., 2021), these improvements do not necessarily extend to higher order cognitive abilities, as shown for example by the lack of effects on distance estimation in favor of individual visuospatial skills (Muffato et al., 2025).

Hence, although Attention Restoration Theory (ART) posits that cognition, specifically attention, can be enhanced in environments with specific characteristics such as natural green spaces, raises the possibility that restorative experiences may influence affective states and perceived cognitive functioning more consistently than objective performance outcomes, although the evidence remains mixed. Integrating subjective and objective measures thus remains essential to fully capture the multifaceted impact of environmental exposure on cognition.

### 1.2 The present study

The present study aimed to investigate the effects of exposure to simulated videos of natural and artistic environments on both subjective measures of Perceived Attentive Efficiency (i.e., *Perceived Working Memory Efficiency, Perceived Sustained Attention, and Perceived Resilience to Interference*) and objective measures of Cognitive Performance, as well as on Perceived Restorativeness. The inclusion of a 2D artistic environment, used as another positively-valenced condition, allowed us to assess whether the cognitive and restorative benefits typically associated with nature derive from its intrinsically restorative features or simply from its greater pleasantness. In line with the ART, we hypothesized that participants exposed to both the natural and the artistic videos would report higher both perceived and objective cognitive efficiency, and experience greater perceived restorativeness compared to those in a control condition.

A further aim of the study was to examine whether the effects of exposure to simulated natural and artistic environments differ across age groups, by comparing outcomes between older adults and young adults. Given that aging is associated with significant declines in attentional control and sustained attention (McAvinue et al., 2012), older adults may be more vulnerable to attentional fatigue and might therefore derive greater benefits from restorative environments, although the extent to which they actually make use of such benefits remains an open empirical question. In detail, we expected that the positive effects on Perceived Attentive Efficiency and objective Cognitive Performance, as well as on Perceived Restorativeness, would be more pronounced in the older adult group compared to the younger participants.

## 2 Methods

### 2.1 Experimental design

A 2x3x2 mixed design was employed in the present study. Repeated measures (i.e., within subjects) were used to test differences in outcome variables across time (T0 vs. T1). The three exposure conditions, i.e., exposure to a virtual 2D natural environment, exposure to a virtual 2D artistic environment, and exposure to a virtual 2D control environment (i.e., office) were included as a between-subjects independent variable. Age groups (young adults vs. older adults) were also included as a between-subjects independent variable to examine age-related differences.

### 2.2 Participants

A total sample of 88 participants took part in the study. Forty-four were young adults, enrolled as follows: 16 (age mean = 22.3; SD = 2.12; Female (F) = 62.5%) in the natural environment exposure, 15 (age mean = 25.7; SD = 4.59; *F* = 80%) in the artistic environment exposure, 13 in the control environment exposure (age mean = 25.7; SD = 5.96; *F* = 76.92%). As well as forty-four were older adults enrolled as follows: 16 in the natural environment exposure (age mean = 68.8; SD = 8.77; *F* = 60%), 15 in the artistic environment exposure (age mean = 64; SD = 9.63; *F* = 46.6%), 13 in the control environment exposure (age mean = 68; SD = 11; *F* = 46.15).

All participants were screened to exclude neurological, psychiatric, or major medical conditions and were volunteers consisting of university students and elderly individuals recruited via the students’ networks. All participants were blind to the hypothesis of study. Prior to enrollment, informed consent was obtained from all participants.

### 2.3 Materials

#### 2.3.1 Stimuli selection

**Figure 1:**
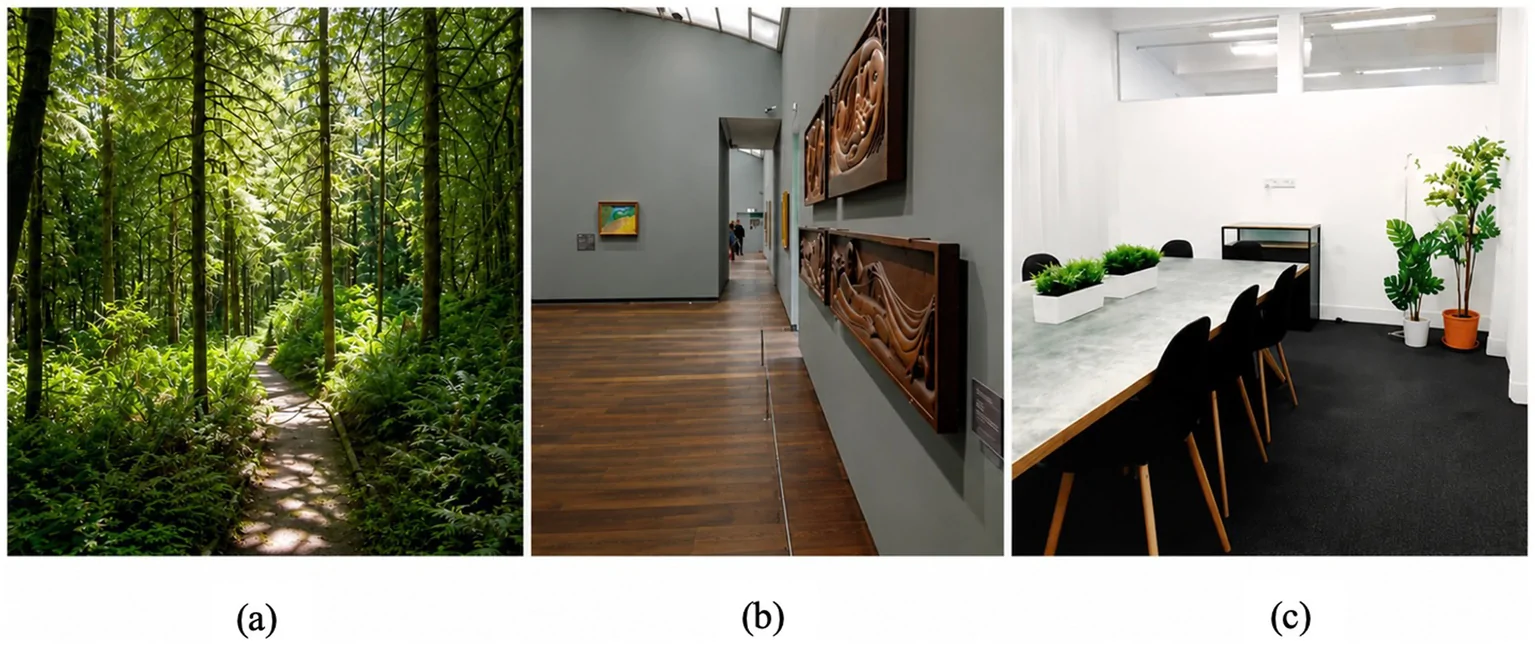
Representative frames illustrating the three video-based exposure conditions: **(a)** virtual 2D natural environment (i.e., forest and hiking paths), **(b)** virtual 2D artistic environment (i.e., Musée d’Orsay, Paris), and **(c)** virtual 2D office environment (i.e., control).

To simulate 2D natural, artistic, and control environments, three 4 K ultra-high-definition videos were collected from YouTube and adapted for the experimental setting. Videos depicting walking in a tropical forest and hiking paths were used for the exposure of natural environment (Figure 1). For the artistic environment exposure, a video of a visit to the Musée d’Orsay in Paris was shown (Figure 1). Lastly, a tour in an indoor office environment was used as a control condition (Figure 1). The brightness of the three videos was adjusted to ensure comparable luminosity and the original audio was retained. Source information for the video stimuli, including links and attribution details, is provided in Supplementary Table S3.

#### 2.3.2 Pre- and post-exposure measures

- We investigated Perceived Attentive Efficiency using three self-report single items inspired by Welsh (2020). Single-item measures were selected to reduce participant burden and limit the length of the pre- and post-assessments, particularly in an older adult sample. Responses were recorded on a Likert scale ranging from 1 (not at all) to 5 (completely), assessing participants’ perceived levels of the following variables: Perceived Working Memory Efficiency, conceptualized as the perceived attentional ability to sustain task-relevant representations despite competing interference (i.e., “Right now, how well do you feel you can hold and recall new information such as details, names, or instructions?”),
- Perceived Sustained Attention, as the perceived ability to maintain attention over time (i.e., “Right now, if you had to start a potentially boring task, how well do you think you could maintain focus on it for an extended period?”),
- Perceived Resilience to Interference, as the degree to which an individual believes they can maintain focus and performance despite distractions or competing stimuli (“Right now, how well do you feel you can ignore distractions like noises, thoughts, or notifications and stay focused?”).

Objective measures of attentive abilities were measured using:

*Flanker Compatibility Test*. The Flanker Compatibility Task (Green and Bavelier, 2003) is a cognitive paradigm designed to evaluate selective attention, response inhibition, and executive control. Participants are presented with a central target stimulus flanked by distractor stimuli, which can be congruent, incongruent, or neutral. In the congruent condition, the flankers match the target, facilitating faster responses. In the incongruent condition, the flanker conflicts with the target, creating cognitive interference that slows reaction times and increases errors. The neutral condition includes non-informative flankers, serving as a baseline for comparison. By analyzing response times and accuracy across these conditions, the task provides insights into attentional filtering and the ability to suppress irrelevant stimuli. The interference effect for errors was computed following the procedure used by Caffarra et al. (2002), and this score was used as outcome in the susbsequent analysis.

*Mackworth Clock Test.* The Mackworth Clock Test (Mackworth, 1968) is a sustained attention task designed to measure vigilance over relatively prolonged periods. Participants observe a circular clock with a moving hand that advances in steps. Occasionally, the hand skips a step, and participants must detect and respond to these rare deviations. Performance is assessed through reaction times and accuracy. The task can be configured based on the frequency of target skips, influencing difficulty and cognitive load. In the frequent condition, skips occur more often, providing regular engagement and reducing fatigue effects. In the infrequent condition, skips are rare, making the task more challenging and increasing vigilance demands over time. D prime (d′) (Mackworth, 1968) was calculated to quantify participants’ sensitivity in detecting target skips. This d′ score was then used as outcome in the subsequent analyses.

*Visual Patterns Test.* The Visual Patterns Test (Della Sala et al., 1997) is a cognitive task designed to assess visuospatial working memory. Participants briefly view a grid containing a specific pattern of filled squares and must recall and reproduce the pattern from memory. As the test progresses, the grid size and pattern complexity increases, placing greater demands on visual working memory. The task can be configured in two ways. In the fixed test condition, participants complete a predetermined set of trials with fixed difficulty levels, allowing for standardized comparisons. In the staircase test condition, difficulty adjusts dynamically based on performance, increasing after correcting responses and decreasing after errors. Memory span for this test was used as outcome in the subsequent analyses.

*Perceived Restorativeness Scale (PRS).* To assess perceived restorativeness post-exposure, the Italian validation of the Perceived Restorativeness Scale - short version (Hartig et al., 1997; Pasini et al., 2014) was used. It is composed of 11 items belonging to four dimensions: Fascination (e.g., “In places like this my attention is drawn to many interesting things”), Being away (e.g., “To get away from things that usually demand my attention I like to go to places like this”), Coherence (e.g., “In places like this everything seems to have its proper place”), and Scope (e.g., “That place is large enough to allow exploration in many directions”). Participants indicated their agreement with statements on a 5-point Likert scale from 1 (strongly disagree) to 5 (strongly agree). Since the scale does not involve a double administration by its nature, the only available comparison is with the responses provided by the other exposure groups. As a limitation, given the cross-sectional nature of the comparison, this instrument poses a greater challenge in interpreting the results.

#### 2.3.3 Procedure

Participants were randomly assigned to one of the three types of exposure (i.e., exposure to natural environment, exposure to artistic environment, and exposure to control environment).

All videos were projected onto a screen measuring 310 cm × 170 cm with a diagonal length of 354 cm, and participants were seated at 240 cm from the screen.

The study took place in two private rooms inside an office building of the local University, and the administration was conducted by two trained researchers. Participants were welcomed into one of the two private rooms. After being informed about the study and filling out the consent form, the pre-exposure measures were administered. Subsequently, in the second room, participants were invited to sit in a chair throughout the duration of the exposure. During this phase participants were invited to watch a 10-min video. At the end of the video, participants were invited to another room, to the administration of the post-exposure measures. The total duration of the study procedure was approximately 50 min.

### 2.4 Statistical analysis

Before conducting both the preliminary and main statistical analyses, the normality and homoscedasticity of the employed variables were checked. Descriptive statistics and preliminary analyses were performed first. A χ^2^ test was performed to examine the association between exposure condition and gender and a Bayesian ANOVA was conducted to examine age differences across the three exposure conditions separately for the younger and older groups.

To address the aims of this study a Bayesian repeated measures 2x3x2 ANOVA was conducted by considering the type of Exposure (i.e., exposure to virtual 2D natural environment, exposure to virtual 2D artistic environment, and exposure to control environment (i.e., control); between subject factor), the Time [i.e., T0 (pre-exposure) and T1 (post-exposure); within-subjects factor], and Age Groups (i.e., young adults vs. older adults; between-subjects factor) as independent variables. Given the aims of the study, we used a Bayesian approach to provide a more informative evaluation of the results than traditional null hypothesis significance testing. In particular, Bayes Factors were used to compare models including the effects of interest with alternative models, allowing us to directly assess the strength of evidence and to distinguish between lack of evidence and evidence in favor of the null hypothesis (Jeffreys, 1961; Lee and Wagenmakers, 2014; Dienes, 2014). Analyses were conducted on both subjective and objective measures. Subjective measures included the three single items assessing Perceived Attentive Efficiency (i.e., Perceived Working Memory Efficiency, Perceived Sustained Attention, and Perceived Resilience to Interference), while objective performance was assessed through the Flanker Compatible Test, the Mackworth Clock Test, and the Visual Patterns Test. Finally, the PRS was included at T1 only. For this outcome, a 3×2 between-subjects design was employed.

All models included the main effects of Time, Exposure, and Age Groups, their interactions, and the combination of both. We identified the best-fitting model and then proceeded to the analysis of effects.

To evaluate the strength of evidence, we adopted the classification by Lee and Wagenmakers (2014), based on Jeffreys (1961) framework. All analyses were conducted using Jamovi software (version 2.3.28; The Jamovi Project, 2022) with the Bayesian (“jsq”) package (Morey and Rouder, 2018; Rouder et al., 2012).

## 3 Results

### 3.1 Descriptive statistics and preliminary analyses

In Table 1, mean and standard deviation for all the variables entered in the analysis together with marginal means for the two assessments - before and after exposition - grouped by Age Groups (young adults and older adults) are shown.

**Table 1:** Mean and standard deviation of selected variable.

	Exposure	Age Groups	Age	PWME pre	PWME post	VPT pre	VPT post	PSA pre	PSA post	MCT pre	MCT post	PRI pre	PRI post	FCT pre	FCT post	PRS post
N	Artistic Env.	Older adults	15	15	15	15	15	15	15	15	15	15	15	15	15	15
		Young adults	15	15	15	15	15	15	15	15	15	15	15	15	15	15
	Natural Env.	Older adults	16	16	16	16	16	16	16	16	16	16	16	16	16	16
		Young adults	16	16	16	16	16	16	16	16	16	16	16	16	16	16
	Control	Older adults	13	13	13	13	13	13	13	13	13	13	13	13	13	13
		Young adults	13	13	13	13	13	13	13	13	13	13	13	13	13	13
Mean	Artistic Env.	Older adults	70.133	3.067	2.933	11.933	11.933	3.800	4.267	2.628	2.994	4.200	4.400	0.042	−0.001	35.400
		Young adults	25.667	4.133	4.200	16.333	16.933	3.867	4.400	4.324	4.506	3.467	3.867	0.025	0.007	29.867
	Natural Env.	Older adults	68.813	2.750	2.625	11.125	12.188	4.000	4.188	3.429	3.370	4.250	4.688	0.037	0.020	34.688
		Young adults	22.313	4.000	4.250	17.875	18.750	3.688	4.063	4.411	4.101	3.625	3.938	0.011	0.006	31.375
	Control	Older adults	69.462	2.692	2.538	8.692	12.692	3.769	3.769	3.048	3.103	4.077	4.154	0.001	0.018	14.769
		Young adults	25.692	4.154	4.000	16.154	18.385	3.615	3.231	4.576	4.894	3.538	3.385	0.012	0.035	20.462
Standard deviation	Artistic Env.	Older adults	9.628	1.033	1.033	1.792	3.900	0.414	0.458	1.361	1.417	0.561	0.507	0.102	0.061	7.059
		Young adults	4.593	0.743	0.941	3.222	2.987	0.640	0.507	0.669	1.092	0.990	1.060	0.060	0.034	6.128
	Natural Env.	Older adults	8.773	0.775	1.088	4.745	5.319	0.816	0.750	1.369	1.311	0.775	0.479	0.099	0.053	8.452
		Young adults	2.120	0.365	1.000	2.918	2.978	0.602	0.998	0.714	0.759	0.806	0.854	0.053	0.050	6.270
	Control	Older adults	10.990	0.855	1.198	6.343	5.105	0.599	0.832	1.725	1.798	0.760	0.899	0.065	0.060	10.670
		Young adults	5.964	0.555	0.816	2.734	4.426	0.650	0.599	0.717	0.822	0.877	1.044	0.045	0.064	11.392

PWM, Perceived Working Memory Efficiency; VPT, Visual Pattern Test; PSA, Perceived Sustained Attention; MCT, Mackworth Clock Test; PRI, Perceived Resilience to Interference; FCT, Flanker Compatibility Test; PRS, Perceived Restorativeness Scale.

Preliminary analyses (i.e., χ^2^ on gender and the Bayesian ANOVA on age across the three exposure) reveled that gender allocation did not significantly differ across the three exposure conditions (χ^2^ (2) = 1.74, *p* = 0.418), as well as the absence of differences of age distribution across exposure conditions in both age groups (BF₁₀ = 0.732 for younger adults; BF₁₀ = 0.062 for older adults).

### 3.2 Bayesian ANOVAs

**Figure 2:**
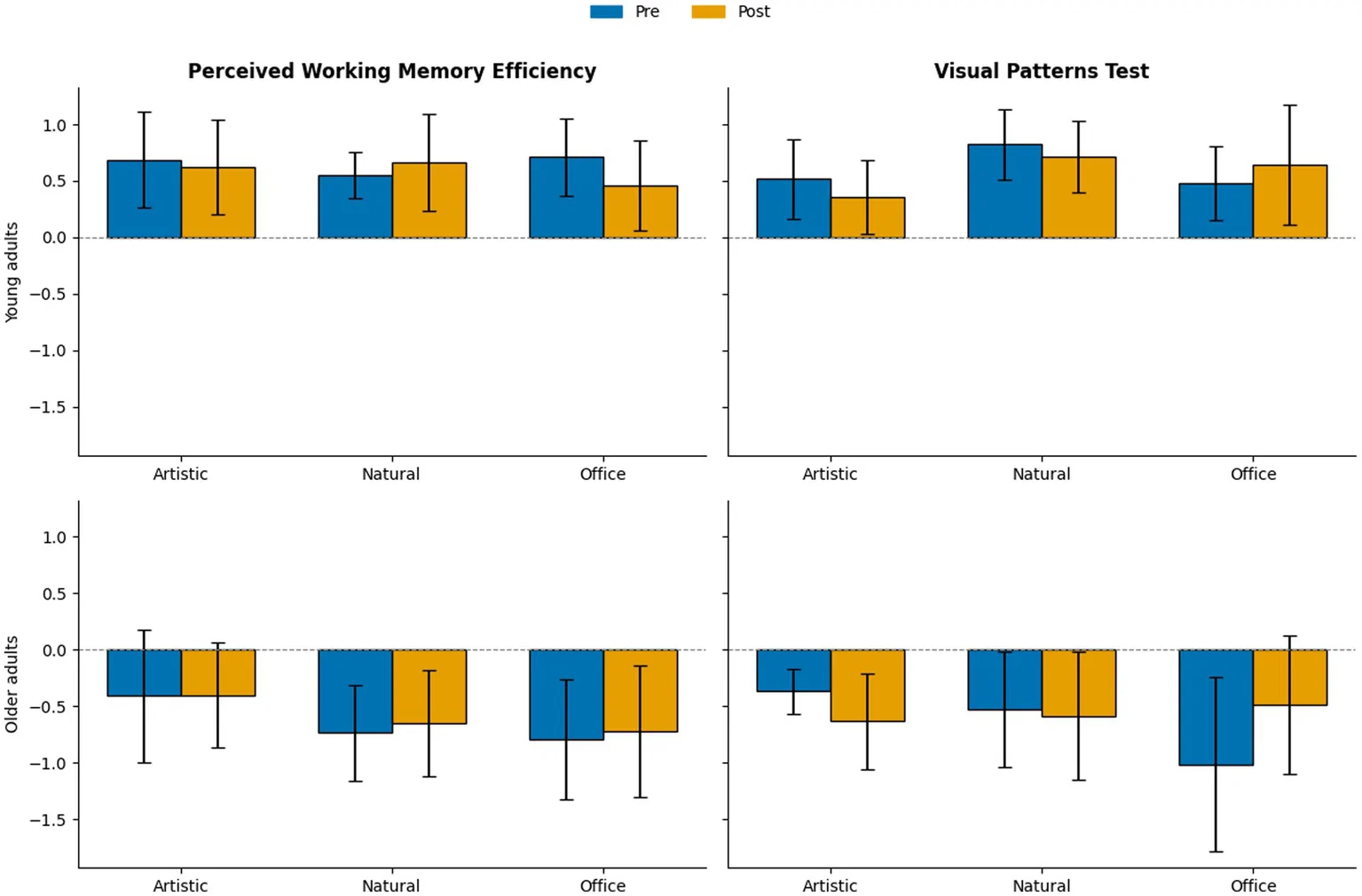
Mean standardized (z-score) pre- and post-test performance on the perceived working memory efficiency (subjective measure; left panels) and the Visual Patterns Test (objective measure; right panels) across exposure conditions (Artistic, Natural, and Office), shown separately for young adults (top row) and older adults (bottom row). Error bars represent 95% confidence intervals.

*Perceived Visuo-spatial Working Memory Efficiency.* Figure 2 older adults reported lower levels of perceived visuo-spatial working memory efficiency than younger adults across both assessment phases. This pattern was consistent at both pre-exposure (older adults: 2.841 ± 0.888; younger adults: 4.091 ± 0.563) and post-exposure (older adults: 2.705 ± 1.091; younger adults: 4.159 ± 0.914), with no substantial changes over time observed within either age group.

The Bayesian model comparison revealed that the best-fitting model included only Age Groups (P(M|data) = 0.609, BF_10_ = 2.829 × 10^9^) (Table 2), indicating extreme evidence in favor of this model compared to the null. Consistently, the analysis of effects (Table 2) provided additional support for this result, revealing extreme evidence in favor of the main effect of Age Groups and supporting its inclusion in the model (P(incl|data) = 1.000, BF_inclusion_ = 1.11 × 10^9^).

**Table 2:** Summary of Bayesian model comparisons and effect analyses across all outcomes.

Outcomes	Model comparison and analysis of effect	BF	Interpretation
Perceived working memory (subjective measure)	*Best-fitting model*		
	Age group	>100	Extreme evidence for H1
	*Analysis of effect*		
	Age group	>100	Extreme evidence for H1
Visual pattern test (objective measure)	*Best-fitting model*		
	Time, age groups and exposure, Time × exposure	>100	Extreme evidence for H1
	*Analysis of effect*		
	Age group	>100	Extreme evidence for H1
	Time	30–100	Very strong evidence for H1
	Time × exposure	1–3	Annecdotical evidence for H1
Perceived sustained attention efficiency (subjective measure)	*Best-fitting model*		
	Time, exposure, Time × Exposure	>100	Extreme evidence for H1
	*Analysis of effect*		
	Time	>100	Extreme evidence for H1
	Exposure	>100	Extreme evidence for H1
	Time × Exposure	30–100	Very strong evidence for H1
Mackworth clock test (objective measure)	*Best-fitting model*		
	Age groups	>100	Extreme evidence for H1
	Analysis of effect		
	Age Groups	>100	Extreme evidence for H1
Perceived resilience to interference efficiency (subjective measure)	*Best-fitting model*		
	Time and age group	>100	Extreme evidence for H1
	*Analysis of effect*		
	Age Groups	>100	Extreme evidence for H1
	Time	10–30	Strong evidence for H1
	Time × Exposure	30–100	Very strong evidence for H1
Flanker compatibility test (objective measure)	*Best-fitting model*		
	Null model	-	-
	*Analysis of effect*		
	No evidence of effect	-	-
Perceived restorativeness (only post exposure)	*Best-fitting model*		
	Exposure	>100	Extreme evidence for H1
	*Analysis of effect*		
	Exposure	>100	Extreme evidence for H1

For each dependent variable (subjective and objective measures), the table reports the best-fitting model identified via Bayesian repeated-measures ANOVA/ANCOVA and the corresponding analysis of effects. Bayes Factors (BF) are provided alongside their interpretation according to Wagenmakers et al. (2018). Evidence levels range from anecdotal to extreme support for the alternative hypothesis (H1).

*Actual Visuospatial Working Memory Efficiency (Visual Pattern Test, VPT)* Figure 2 younger adults performed better than older adults on the VPT across both assessment phases. Additionally, for many participants, performance generally improved from pre- to post-exposure across conditions. Although participants exposed to the office control environment appeared to show greater improvement over time, supplementary analyses indicated that this pattern reflected baseline differences between groups rather than exposure-related effects (Supplementary Tables S1, S2).

The Bayesian model comparison Table 2) revealed that the best-fitting model included the main effect of Time, Age Groups and Exposure, and the interaction Time × Exposure (P(M|data) = 0.310, BF_10_ = 1.95 × 10^10^), providing the extreme evidence in favor of this model compared to the null. The analysis of effect (Table 2) revealed extreme evidence in favor of including the main effect of Age Groups (P(incl|data) = 1.000, BF_inclusion_ = 2.05 × 10^8^). Very strong evidence also supported the inclusion of the main effect of Time (P(incl|data) = 0.991, BF_inclusion_ = 40.907). Lastly, anecdotal evidence for the interaction between Time × Exposure (P(incl|data) = 0.539, BF_inclusion_ = 2.532) was provided.

**Figure 3:**
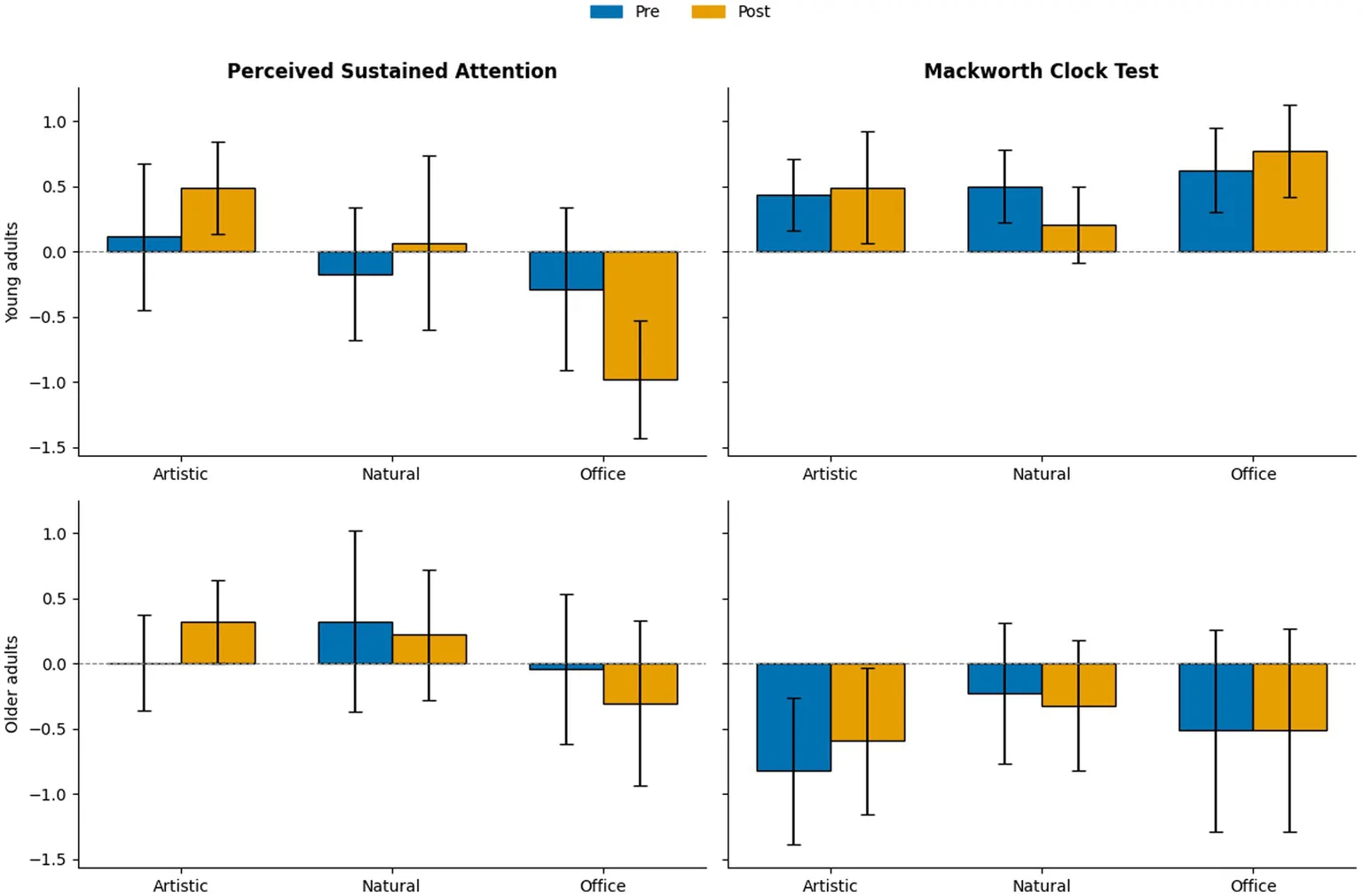
Mean standardized (z-score) pre- and post-test performance on the perceived sustained attention (subjective measure; left panels) and the Mackworth Clock Test (objective measure; right panels) across exposure conditions (Artistic, Natural, and Office), shown separately for young adults (top row) and older adults (bottom row). Error bars represent 95% confidence intervals.

*Perceived Sustained Attention Efficiency* (Figure 3). Changes in perceived sustained attention differed across exposure conditions. Specifically, most participants exposed to the natural and artistic environments reported higher levels of perceived sustained attention in the post-exposure phase compared with pre-exposure (natural: pre = 3.844 ± 0.723, post = 4.125 ± 0.871; artistic: pre = 3.833 ± 0.531, post = 4.333 ± 0.479). In contrast, participants in the control environment showed a slight descriptive decrease from pre-exposure (3.692 ± 0.618) to post-exposure (3.500 ± 0.762).

The Bayesian model comparison (Table 2) indicated that the best-fitting model included Time, Exposure, and their interaction (Time × Exposure) (P(M|data) = 0.514, BF10 = 1828.398), providing extreme evidence compared with the null model. The analysis of effects (Table 2) revealed extreme evidence supporting the inclusion of the main effects of Time (P(incl|data) = 0.997, BF_inclusion_ = 120.730) and Exposure (P(incl|data) = 0.997, BF_inclusion_ = 109.486). Importantly, strong evidence supported the inclusion of the Time × Exposure interaction (P(incl|data) = 0.975, BF_inclusion_ = 85.985), indicating that changes over time differed across exposure conditions.

However, further Bayesian analyses revealed no support for a pre-post change in the control condition, despite the descriptive decrease observed in Figure 3 (P(M|data) = 0.380; BF10 = 0.613), suggesting that perceived sustained attention remained substantially stable over time in the neutral environment.

*Actual Sustained Attention Efficiency (Mackworth Clock Test)* Figure 3 younger adults consistently performed better than older adults on the Mackworth Clock Test across both assessment phases. This pattern was evident at both pre-exposure (older adults: 3.044 ± 1.484; younger adults: 4.430 ± 0.691) and post-exposure (older adults: 3.163 ± 1.477; younger adults: 4.473 ± 0.940), with no substantial changes over time observed within either age group.

The Bayesian model comparison (Table 2) revealed that the model including only Age Groups was the best-fitting model (P(M|data) = 0.548, BF10 = 1.19 × 10^5^), providing extreme evidence compared with the null model. Consistently, the analysis of effects (Table 2) showed extreme evidence supporting the inclusion of Age Groups in the model (P(incl|data) = 1.000, BF_inclusion_ = 4.98 × 10^4^), indicating lower sustained attention performance among older adults relative to younger adults across both phases.

**Figure 4:**
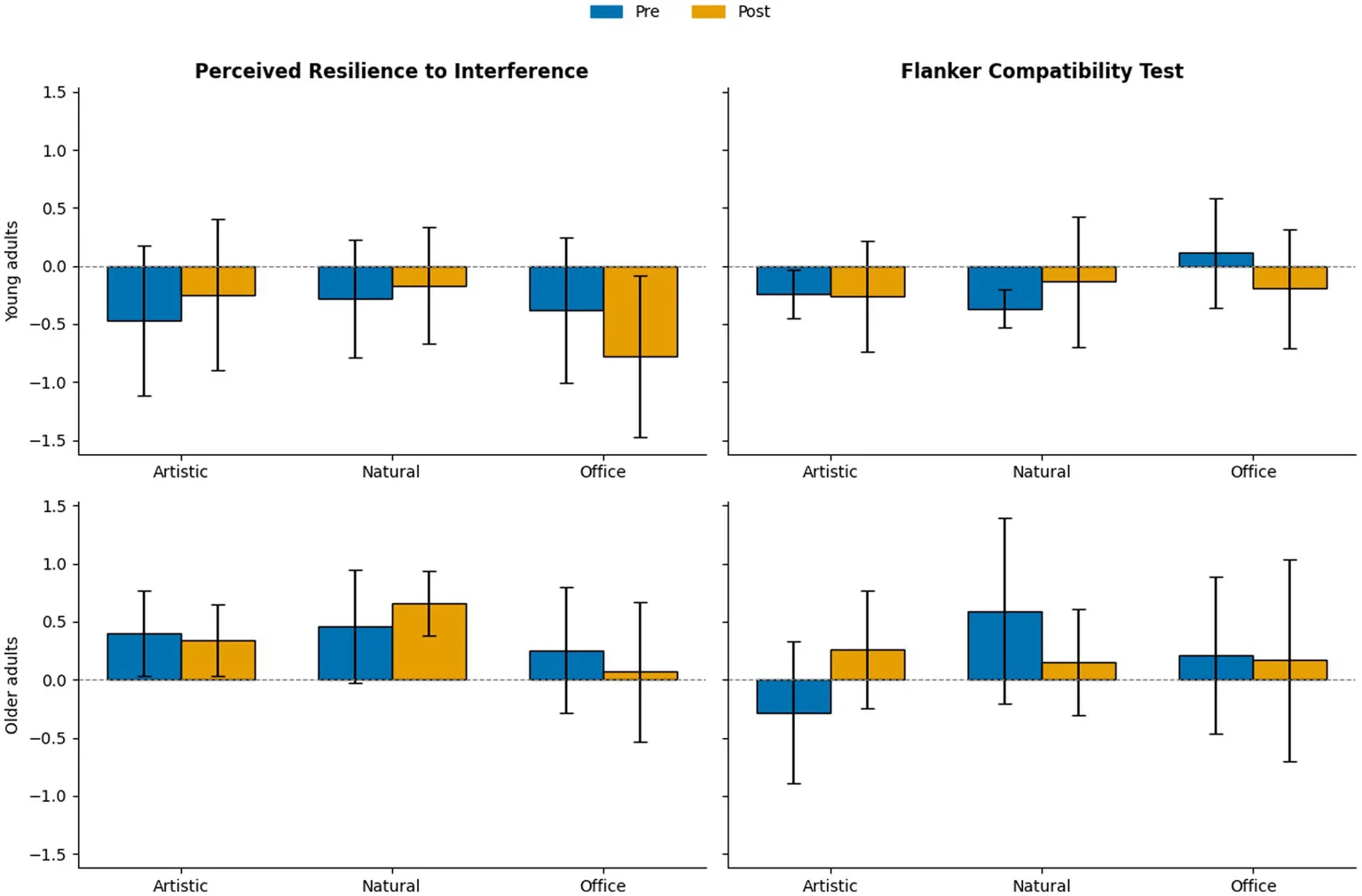
Mean standardized (z-score) pre- and post-test performance on the perceived resilience to interference (subjective measure; left panels) and the flanker compatibility test (objective measure; right panels) across exposure conditions (artistic, natural, and office), shown separately for young adults (top row) and older adults (bottom row). Error bars represent 95% confidence intervals.

*Perceived Resilience to Interference Efficiency.* Figure 4 older adults generally reported higher levels of perceived resilience to interference than older adults across assessment phases. Generally, perceived resilience to interference appeared to increase from pre- to post-exposure across conditions, with descriptively increases observed in the natural and artistic environments than in the control condition (natural: pre = 3.938 ± 0.840, post = 4.313 ± 0.780; artistic: pre = 3.833 ± 0.874, post = 4.133 ± 0.780; control: pre = 3.808 ± 0.849, post = 3.769 ± 1.032).

The Bayesian model comparison (Table 2) revealed that the best-fitting model included both Time and Age Groups (P(M|data) = 0.304, BF₁₀ = 5430.943), providing extreme evidence compared with the null model. The analysis of effects (Table 2) showed extreme evidence supporting the inclusion of the main effect of Age Groups (P(incl|data) = 0.997, BF_inclusion_ = 131.085), Strong evidence also supported the inclusion of the main effect of Time (P(incl|data) = 0.978, BF_inclusion_ = 15.947).

Only anecdotal evidence supported the inclusion of the Time × Exposure interaction (P(incl|data) = 0.376, BF_inclusion_ = 1.303). Therefore, although descriptively, participants exposed to the natural and artistic environments appeared to show greater post-exposure improvements, this pattern should be interpreted cautiously.

*Actual Resilience to interference Efficiency (Inhibitory Control, Flanker Compatibility Test)* Figure 4 overall, no substantial differences were observed across assessment phases, exposure conditions, or age groups. Descriptively, FCT scores remained relatively stable from pre- to post-exposure across all groups, including older adults in the artistic (pre = 0.042 ± 0.102, post = 0.001 ± 0.061), natural (pre = 0.037 ± 0.099, post = 0.020 ± 0.053), and control conditions (pre = 0.001 ± 0.065, post = 0.018 ± 0.060), as well as younger adults in the artistic (pre = 0.025 ± 0.060, post = 0.007 ± 0.034), natural (pre = 0.011 ± 0.053, post = 0.006 ± 0.050), and control conditions (pre = 0.012 ± 0.045, post = 0.035 ± 0.064). The Bayesian model comparison (Table 2) showed that the null model was the best-fitting model (P(M|data) = 0.588, BF₁₀ = 1.000). The analysis of effects (Table 2) revealed that no evidence supported the inclusion of any main effects or interactions.

**Figure 5:**
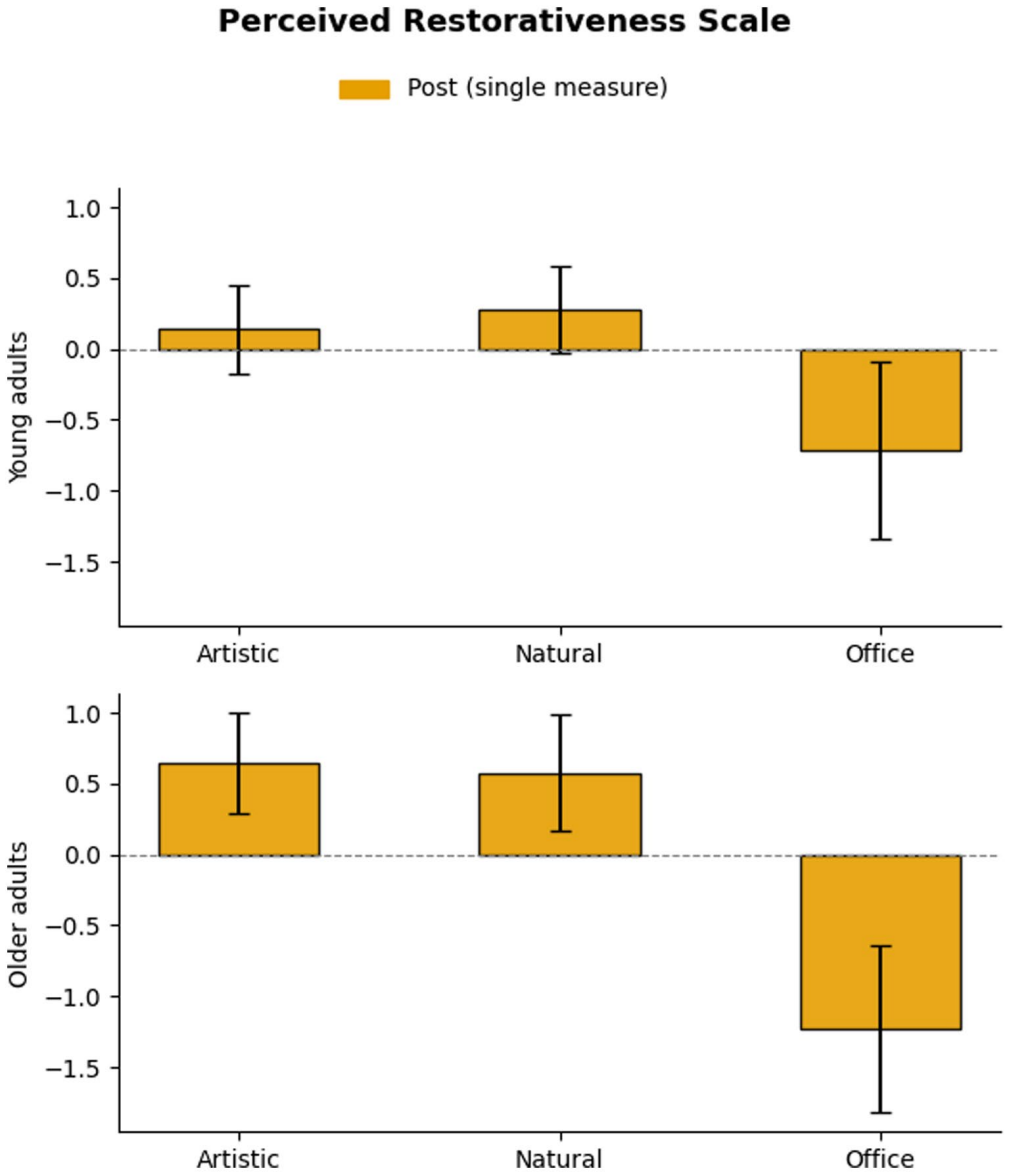
Mean standardized (z-score) pre- and post-test performance on the perceived restorativeness scale after intervention (T1) across exposure conditions (artistic, natural, and office), shown separately for young adults (top row) and older adults (bottom row). Error bars represent 95% confidence intervals.

*PRS (Perceived Restorativeness).* (Figure 5). Participants exposed to the natural and artistic environments reported substantially higher levels of perceived restorativeness than those exposed to the control condition (natural: 33.031 ± 7.511; artistic: 32.633 ± 7.078; control: 17.615 ± 11.197). Because perceived restorativeness was assessed only after the experimental manipulation, analyses focused exclusively on post-exposure differences across conditions.

The Bayesian model comparison (Table 2) indicated that the model including Exposure was the best-fitting model (P(M|data) = 0.666, BF₁₀ = 3.21 × 10^7^), providing extreme evidence compared with the null model. Consistently, the analysis of effects (Table 2) showed extreme evidence supporting the inclusion of Exposure in the model (P(incl|data) = 1.000, BF_inclusion_ = 2.54 × 10^7^).

## 4 Discussion

The present study examined the relationship between exposure to simulated restorative environments (i.e., natural and artistic) and both subjective and objective indicators of cognitive functioning. Benefits emerged only in comparison to the control condition. Contrary to our initial hypotheses and to some previous findings (e.g., Berto, 2005; Berman et al., 2008; Rhee et al., 2024), exposure to 2D natural and artistic environments was not associated with measurable improvements in objective attentional performance under the present experimental conditions. In contrast, participants exposed to these environments reported higher levels of Perceived Attentive Efficiency and Perceived Restorativeness. Taken together, these findings suggest that subjective and objective outcomes may not necessarily change in parallel following brief exposure to simulated restorative environments. While participants exposed to 2D natural and artistic environments reported improvements in Perceived Sustained Attention and Perceived Resilience to Interference, the corresponding objective measures (Mackworth Clock Test and Flanker Compatibility Test, respectively) showed no analogous improvements. This pattern of results aligns with recent evidence suggesting that the restorative effects of environments, both virtual and real, may be primarily affective and perceptual rather than cognitive in nature (in terms of mental representation in Muffato et al., 2025). We can speculate that exposure to pleasant and restorative environments may influence individuals’ beliefs about their cognitive capabilities, creating a subjective sense of improvement that does not necessarily translate into objective performance gains. This may suggest that perceived benefits could be mediated by self-evaluative processes rather than actual improvements in attentional capacities.

While the Attention Restoration Theory (ART) posits that natural environments should restore depleted attentional resources and improve cognitive performance, our results, along with a number of systematic reviews (e.g., de Keijzer et al., 2016; Spano et al., 2023; Ricciardi et al., 2022; Li et al., 2021a,b; Vella-Brodrick and Gilowska, 2022), suggest that these effects may be more context-dependent than originally proposed. The consistent pattern of subjective improvements without corresponding objective performance gains suggests that, within the present study, ART-related outcomes may be more closely reflected in subjective rather than objective measures.

This finding suggests the need for further investigation; however, the novelty of this study lies in considering the possibility that the findings may inform future refinements of the theory in the context of simulated environmental exposure. Experiencing restoration, psychological well-being and perceiving oneself as cognitively effective hold substantial intrinsic value, which should not be overlooked but rather emphasized, especially in vulnerable groups such as older adults. Indeed, the perception of well-being can play a decisive role at this stage of life. Nevertheless, this positive perception may also have a potential downside when it leads individuals to underestimate their limitations. This finding raises the possibility that, in some contexts, increases in perceived cognitive functioning may not correspond to objective performance changes. One possible implication of this subjective-objective dissociation is that perceived improvements may not always correspond to measurable changes in performance. Whether such discrepancies influence everyday decision-making remains an open empirical (Siciliano et al., 2021). At the same time, we acknowledge that higher subjective well-being has been associated with better quality of life and, in the long term, with more favorable health outcomes (Liu et al., 2023). Thus, an important open question is whether, and under which conditions, perceived improvements serve as a protective factor or, conversely, as a source of reduced self-monitoring that may increase vulnerability in everyday life.

Subsequently, a particularly noteworthy aspect of our findings concerns the comparable effects of natural and artistic environments on both subjective measures and perceived restorativeness. Both conditions produced similar improvements in Perceived Sustained Attention and Perceived Resilience to Interference compared to the control condition, and both environments were rated as equally restorative on the Perceived Restorativeness Scale. This pattern suggests that the restorative benefits typically attributed to natural environments may not be exclusively tied to their *natural* characteristics but may instead derive from their *positive valence*, aesthetic appeal, or capacity to engage attention in a pleasant manner, indicating that the enjoyment of art as a pleasurable experience (Mauri et al., 2023) can represent a valid alternative to simulated natural environments. The possibility of achieving comparable benefits through artistic stimuli expands the range of options for digital interventions, urban planning, and institutional settings such as schools, workplaces, or healthcare facilities. In practical terms, incorporating art-based or aesthetically appealing virtual content may provide accessible, low-cost opportunities to foster subjective attentional restoration across diverse populations. Beyond their aesthetic qualities, art based virtual environments such as museums may also provide a richer stream of information and novelty, which can be intrinsically rewarding and cognitively engaging. This “information reward” mechanism may boost interest and arousal, in contrast with the more calming and soothing effects often attributed to green environments, suggesting that different types of virtual settings might support well-being through partially distinct pathways.

Our results confirmed age-related differences, with older adults showing lower cognitive performance and perceived working memory efficiency than younger adults (Burmester et al., 2016). However, environmental exposure did not yield differential restorative effects by age, contrary to our hypothesis. Although older adults might be especially sensitive to such effects due to changes in attention and executive function (Kalantari et al., 2022), our findings did not support this.

While it offers valuable insights, this study has a number of limitations that need to be acknowledged. The use of 2D video presentations rather than immersive 3D virtual reality may have limited the magnitude of effects observed in this study. For this reason, the present findings should be interpreted in light of the methodological characteristics of the study, which relied on simulated 2D environments rather than direct exposure to real settings. Previous research has highlighted the importance of presence and immersion in virtual environments (Liszio et al., 2018; Li et al., 2021a), suggesting that more immersive technologies might produce stronger restoration effects. However, the 2D format used in this study offers practical advantages reduced motion sickness risk (particularly relevant for older adult participants given the documented issues with VR in this population; Howard and Van Zandt, 2021), and broader applicability in real-world settings. Indeed, this choice was primarily driven by the greater feasibility and scalability of methods based on 2D technologies that are potentially accessible in most homes. This is particularly crucial from a translational perspective, as the goal is to move from laboratory research to broad-scale implementation. Moreover, recent research actually downplays the role of mere technological immersion in sense of presence (e.g.: people may feel strong sense of presence within a desktop video game thanks to an engaging narrative) (Triberti et al., 2025). Future research may feature the measurement of sense of presence as associated with VR effectiveness, and also explore different results based on virtual environments that are designed according to users’ needs and preferences (Pizzoli et al., 2019).

An additional limitation concerns the selection of the video stimuli. Although the environments were chosen to represent distinct categories (i.e., natural, artistic, and control environments), their restorative potential was not formally validated prior to the experiment. Future studies may benefit from preliminary validation procedures to better characterize the restorative properties of the selected stimuli.

Although the 10-min exposure aligns with previous studies (Wilkie et al., 2023), it may not have been sufficient to reliably elicit measurable cognitive improvements under the present conditions. Similarly, this decision was motivated by a commitment to ensuring large-scale applicability. Studies indicate that prolonged, passive, video exposure can lead to fatigue, boredom, and high dropout rates (e.g., Huang et al., 2023). Evidence regarding the influence of exposure duration (≤5 min, 5–10 min, ≥10 min) on outcomes is limited (Bolouki et al., 2025). Exposure protocols vary considerably, ranging from brief to extended 30-min sessions, with shorter exposures predominantly affecting affective states (Spano et al., 2023); however, precise recommendations for optimal duration remain undefined. Moreover, emerging evidence suggests that effect sizes may vary with duration, with brief exposures (e.g., 5–10 min) often sufficient to improve affective and psychophysiological outcomes (Suppakittpaisarn et al., 2023), whereas longer or repeated sessions are more likely to elicit cognitive benefits, although findings remain inconsistent (Spano et al., 2023;). Our study employed a brief exposure protocol within this common range, which may partially account for the observed pattern in which subjective restoration effects were more evident than objective cognitive changes. Accordingly, the absence of measurable cognitive improvements in the present study should be interpreted cautiously, as cognitive restoration effects may require longer or repeated exposure to emerge.

In addition, baseline differences between exposure groups were observed for one cognitive outcome, the Visual Pattern Test. Although this represents a limitation and warrants caution in interpreting cognitive findings, the groups were otherwise comparable on the remaining cognitive measures. Where baseline differences in age were identified, we adjusted for age as a covariate in our analyses (ANCOVA), which helps to control for the potential confounding effect of age and mitigates concerns about baseline imbalance. Lastly, perceived restoration was assessed only at post-exposure, as this construct is inherently defined as a response to the exposure experience.

Several key research priorities emerge from these findings. First, continued investigation into the mechanisms underlying the subjective-objective dissociation in environmental restoration effects is essential for advancing theoretical understanding. Second, the development and validation of more sensitive cognitive measures that capture the specific aspects of attention and cognitive control most susceptible to environmental restoration represents a critical methodological advancement. Traditional cognitive tasks may not adequately assess the domains most responsive to environmental manipulation.

Third, systematic research examining optimal parameters for virtual environmental interventions, including duration, frequency, timing, and level of immersion, would inform the development of evidence-based protocols for clinical and therapeutic applications.

## 5 Conclusion

The present study suggested that exposure to simulated 2D natural and artistic environments was associated with improvements in subjective perceptions of cognitive functioning and restorativeness, while suggesting a potential dissociation between subjective experiences and objective cognitive performance measures. The observed subjective-objective dissociation may encourage further investigation into how the predictions of Attention Restoration Theory are reflected across subjective and objective outcomes, particularly in simulated 2D environments. From a physiological perspective, ART may be consistent with theoretical accounts proposing reduced demands on effortful attention. These theoretical accounts may help interpret why experiential outcomes such as perceived restoration and positive affect are more consistently observed than changes in higher-order cognitive abilities.

Despite the absence of objective cognitive improvements, the findings simultaneously underscore the value of subjective restoration experiences for psychological health and well-being. The consistent benefits observed across different environmental types support continued investigation and application of virtual restorative environments, particularly for populations with limited access to natural environments, including hospitalized patients, institutionalized older adults, and individuals with mobility constraints. The comparable pattern of subjective outcomes observed for 2D natural and artistic environments may offer useful directions for future intervention design, allowing for greater personalization based on individual preferences and cultural backgrounds, which could enhance the acceptability and effectiveness of virtual environment interventions in clinical and therapeutic settings. Additionally, the low technological requirements and the limited duration of exposition suggest that even an exposure like the one developed and used in this study could provide potentially useful support to individuals interested in engaging with natural or artistic stimuli but who, for a variety of reasons, do not have the full opportunity to do so. The subjective feeling of having restored one’s own attentional and cognitive resources may still hold significant value for psychological well-being and quality of life. Although subjective perceptions of restoration should not be interpreted as evidence of objective cognitive improvement, they may nevertheless reflect a valuable sense of relief or reduced mental fatigue following exposure to restorative environments. Future research is needed to better understand the relationship between subjective restoration experiences and measurable cognitive outcomes.

## Data Availability

The data analyzed in this study is subject to the following licenses/restrictions: data will be made available upon request. Requests to access these datasets should be directed to luigi.tinella@unipegaso.it.
