# Supplementary material for: Differential effects of simulated restorative environments on subjective and objective attentional outcomes
Source: Front Psychol. 2026 Jul 10;17:1791175. doi: 10.3389/fpsyg.2026.1791175 (PMC13395733; doi:10.3389/fpsyg.2026.1791175)
Supplement: Supplementary file 1 [file Supplementary_file_1.docx]

| **Table S1.** Model comparison of Bayesian ANCOVA with Visual Pattern Test as outcome. | | | | | | | | | | | |
| --- | --- | --- | --- | --- | --- | --- | --- | --- | --- | --- | --- |
| **Models** | | **P(M)** | | **P(M\|data)** | | **BF_M_** | | **BF_10_** | | **error %** | |
| Null model |  | 0.125 |  | 0.000 |  | 0.000 |  | 1.000 |  |  |  |
| VPT_T0 |  | 0.125 |  | 0.189 |  | 1.626 |  | 27152304154046.055 |  | 0.007 |  |
| Exposure |  | 0.125 |  | 0.000 |  | 0.000 |  | 0.047 |  | 0.014 |  |
| VPT_T0+ Exposure |  | 0.125 |  | 0.172 |  | 1.453 |  | 24760434782048.500 |  | 1.991 |  |
| Age Groups |  | 0.125 |  | 0.000 |  | 0.000 |  | 2159825.365 |  | 0.000 |  |
| VPT_T0+ Age Groups |  | 0.125 |  | 0.401 |  | 4.685 |  | 57745715306195.617 |  | 0.824 |  |
| Exposure + Age Groups |  | 0.125 |  | 0.000 |  | 0.000 |  | 123445.832 |  | 1.568 |  |
| VPT_T0n + Exposure + Age Groups |  | 0.125 |  | 0.239 |  | 2.194 |  | 34375559716248.156 |  | 1.229 |  |
|  | | | | | | | | | | | |

| **Table S2.** Analysis of effects Bayesian ANCOVA with Visual Pattern Test as outcome. | | | | | | | |
| --- | --- | --- | --- | --- | --- | --- | --- |
| **Effects** | | **P(incl)** | | **P(incl\|data)** | | **BF_Inclusion_** | |
| VPT_T0 |  | 0.500 |  | 1.000 |  | 63082277.712 |  |
| Exposure |  | 0.500 |  | 0.411 |  | 0.697 |  |
| Age Groups |  | 0.500 |  | 0.640 |  | 1.775 |  |
|  | | | | | | | |

**Table S3**. Source information for video stimuli.

| **Condition** | **Description** | **Source** | **URL** |
| --- | --- | --- | --- |
| Natural environment | Walking in tropical forest and hiking paths | YouTube video | <https://www.youtube.com/watch?v=oSmUI3m2kLk> |
| Artistic environment | Visit to the Musée d'Orsay in Paris | YouTube video | <https://www.youtube.com/watch?v=u9TPIxBZGTw> |
| Control environment | Indoor office | YouTube video | <https://www.youtube.com/watch?v=OQQ4a4Os6cE> |
